# The Normative Power of Consent and Limits on Research Risks

**DOI:** 10.1007/s10677-024-10441-4

**Published:** 2024-05-22

**Authors:** Aaron Eli Segal, David S. Wendler

**Affiliations:** Department of Bioethics, Clinical Center, National Institutes of Health, Bethesda, MD 20892 USA

**Keywords:** Ethics, Kidney Biopsy, Altruistic, Donor, Research

## Abstract

Research regulations around the world do not impose any limits on the risks to which consenting adults may be exposed. Nonetheless, most review committees regard some risks as too high, even for consenting adults. To justify this practice, commentators have appealed to a range of considerations which are external to informed consent and the risks themselves. Most prominently, some argue that exposing consenting adults to very high risks has the potential to undermine public trust in research. This justification assumes that it is not the magnitude of the risks themselves which raises concern, but the way in which the public might respond to them. This justification thus depends on the possibility that the public will find out about the risks and respond to them in the specified way. Like the other proposed external justifications, it thereby fails to offer a reason to think that exposing consenting adults to very high risks is problematic in itself. In the present paper, we describe and endorse a different justification. Rather than appealing to external factors, we argue that limits on risks for consenting adults trace to internal limits on informed consent, to limits on the things consent can and cannot make ethically permissible. In doing so, we aim to provide a firmer conceptual basis for the view that some research risks are unacceptably high, no matter how the research is conducted.

Some clinical trials are unethical because the ‘net’ risks—the risks which exceed the trials’ potential to benefit participants—aren’t justified by the trials’ potential to benefit others. (Wendler and Miller 2007) Other trials are unethical because the net risks are too high, independent of the trials’ potential to benefit others. This condition raises the question of which net risks are acceptable, and which ones are excessive in the context of medical research.

Some commentators argue that there are no absolute limits on the risks to which participants may be permissibly exposed. (Eyal 2020; Steel 2019) According to utilitarians, for example, clinical trials are ethically appropriate as long as the net risks are justified by the trials’ potential to benefit others, and there are no less risky ways to realize the benefits in question. On this view, any level of net risks can be acceptable, no matter how high, provided it is justified by trials’ potential to benefit others.

Guidelines around the world reject this approach when it comes to individuals who cannot consent. For example, the Council for International Organizations of Medical Sciences (CIOMS) guidelines permit adults who cannot consent to be enrolled in research that does not offer the potential for participant benefit only when the risks are minimal, or the risks are a minor increase over minimal and the research has compelling social value.^1^

In contrast, most regulations do not impose any limits on the net risks to which consenting adults may be permissibly exposed.^2^ Some argue that this is as it should be—no research risks are so high that imposing them on consenting participants is necessarily impermissible. (Eyal 2020, Steel 2019) According to libertarians, for instance, prohibiting competent adults from exposing themselves to some risks represents an unacceptable infringement on their right to determine what they do with their bodies. (Shaw 2014) Most review committees and commentators reject this approach, holding that some research risks are impermissibly high, even for consenting adults. (Miller and Joffe 2009; London 2009)

We agree. Imagine, for instance, a study with a 50% risk of death that enrolls 10 competent adults, and has a 1 in 10,000 chance of identifying an intervention which would incrementally improve treatment for a common illness. If the number of potential beneficiaries is sufficiently high, the social value of this study could well outweigh these risks. Nonetheless, such a study would be ethically unacceptable, first and foremost because it imposes excessive risks on participants.^3^

To try to justify this practice, commentators have appealed to a range of considerations, including public trust in medical research and the role participants play in research. According to these accounts, the limits on what participants can consent to derive from factors external to consent and the risks themselves. On the public trust justification, it is not the magnitude of the net risks that makes the research unacceptable. It is the way the public might respond to them. These justifications are therefore contingent and unstable. Justifying risk limits on the grounds that exposing participants to very high risks has the potential to undermine public trust depends on the possibility that a sufficient number of people will learn about the trial and respond in the specified way. This justification thus suggests that the same level of risks could be acceptable in a trial that is conducted in private, or at a time when the public is distracted by world events, such as a war or a pandemic.^4^

These justifications also do not yield a method for determining which risks are excessive. For example: which risks are sufficiently high that they might undermine public trust? In light of this uncertainty, stakeholders tend to rely on their intuitions regarding which risks are acceptable, and which are excessive. The problem with this approach is that individuals have widely divergent intuitions regarding which risks are acceptable. Moreover, intuitive judgments of risk are subject to a number of cognitive biases that do not track the magnitude of the risk in question. (Tversky and Kahneman 1974; Slovic 1987) For example, unfamiliar activities are frequently judged to be riskier than familiar ones, a particularly problematic bias in the context of research that often involves novel interventions.

Alternatively, or in conjunction with reliance on intuition, guidelines and stakeholders sometimes evaluate research risks by comparing them to the risks of other activities. Most prominently, minimal risks are frequently defined as risks that do not exceed the risks that ‘individuals ordinarily encounter in daily life.’^5^ The idea here is that participation in research that poses net risks involves accepting risks in order to benefit others. Hence, what level of net risks is acceptable can be estimated by comparison to other activities that impose risks on competent adults for the benefit of others, such as living organ donation and firefighting. (Miller and Joffe 2009; London 2009)

This approach ensures that the risks we permit in the context of clinical research are not significantly greater than those we permit in other contexts. It thus ensures that research participants are not treated worse in this regard. However, this approach does not provide a justification for why the risks permitted in these comparator altruistic activities are, in fact, permissible. They also do not provide a justification for why the risks that *aren’t* permitted in the comparator activities are, in fact, impermissible. In what follows, we attempt to address this gap in the literature by offering an account of why risk limits, even for consenting adults, are justified, and how these limits can be determined. This argument is based on the normative significance of informed consent. Rather than appealing to external factors, such as public trust, we will argue that risk limits for consenting adults can be explained as internal limits on informed consent itself, on the kinds of things that consent can and cannot make ethically permissible.^6^

We argue that this approach vindicates the assumption that there are limits on the research risks that can be permissibly imposed on consenting adults. The present approach thereby offers a clear response to those who maintain that any limits on the risks to which competent adults may consent are inappropriate. This is not to suggest that appeal to moral theory alone can determine precisely where the limits are. Moral theory alone cannot, for instance, determine whether the limit on an acceptable risk of death in consenting adults is 1 in 8,000 or 1 in 10,000. Making that determination requires the judgment of reasonable people, based on the relevant facts and with an understanding of the circumstances. What moral theory can provide is an account of why there are limits on research risks and what considerations are relevant to determining where those limits lie.

Section 1: The Normative Power of Consent

To begin, consider the limits on the net risks of research with participants who *cannot* consent (e.g. children, adults with severe dementia). These limits are justified on the grounds that the individuals are less able or completely unable to protect their own interests. They therefore merit significant protections which go beyond those that apply to consenting adults. To this end, most guidelines and regulations limit research with participants who cannot consent to minimal, or a minor increase over minimal net risks.^7^

The fact that individuals who cannot consent may be permissibly exposed to these risks implies that it can be permissible to expose consenting adults to higher risks. If that were not the case, these standards would fail to offer *additional* protections for individuals who cannot consent. Moreover, individuals who cannot consent merit *significant* added protection. This suggests that it can be acceptable to expose competent adults to net risks that are significantly higher than a minor increase over minimal. But how much higher? To answer that question, we need to determine how consent justifies exposing consenting adults to risks, and on that basis, how much risk it can justify.

Consent is a normative power. (Thomson 1992; Wellman 1995, 1997; Wertheimer 2000; Shiffrin 2008; Watson 2009; Owens 2012; Dempsey 2013; Enoch 2014; Dougherty 2015; Manson 2016; Koch 2018) That is, consent is a power or ability to change normative facts, or to change what is and what is not permissible. Examples of normative powers include not only consent, but also commands, legislation, promises, and contracts. Consent differs from these other types of normative powers first and foremost because consent makes someone’s action permissible by waiving one’s right against them that they not perform the action consented to. In this way, consent releases someone from a directed duty by waiving one’s right against them. In the bioethics literature on consent, there is relatively robust agreement that the power of consent is exercised just in case an individual’s attempt to waive their right is competent, informed, based on an understanding of relevant information, voluntary, and involves a token communication of consent.^8^ (Faden and Beauchamp 1986; Beauchamp and Childress 2019; Millum and Bromwich 2021)

To consider a related normative power, when A *promises* to pick B up at the airport, A changes the normative facts, making it morally required for them to pick B up at the airport, where doing so was previously morally optional. Similarly, when A consents to B entering their home, A changes the status of B’s entering their home from impermissible to permissible.

At the same time, consent cannot change just any normative facts. There are limits. We cannot, for example, make torture permissible by agreeing to it. Consent cannot make saving the lives of others impermissible. So what explains the fact that individuals can change the normative status of others’ actions by consenting, and what are the scope and limits of this power?

The literature offers two different answers. The first claims that we have some normative power when it is in our interests to have it (Especially Raz 1972 and Owens 2012). The second claims that we have some normative power when our possession of it is intimately bound up with being autonomous agents, or when it is partly constitutive of being autonomous. (Especially Thomson 1992, Hurd 1996, Shiffrin 2008, Chang 2013) In the words of one proponent of the autonomy-based view, “to respect persons as autonomous is to recognize them as the givers and takers of rights and duties. It is to conceive of them as very powerful moral magicians.” (Hurd 1996, 124).

Both approaches are plausible, and both have been supported by strong arguments. Hence, rather than try to choose between them, we will consider the implications each has for setting risk limits on research with competent adults. We start with the interests-based approach, and then turn to the autonomy-based approach.

Section 2: How Consent Justifies Risks: Interests

We have a strong interest in being able to interact with others. Doing so makes our lives go better for our own sakes—we are able to achieve more through cooperation than alone, and we are able to enjoy the fruits of others’ company. But interacting with others also poses risks; it can lead to their becoming involved in our lives in ways that are problematic for us. Having control over how and when we interact with others offers the means to realize the benefits while minimizing the risks. This explains why having the normative power of consent is in our interests.

To see this, consider again A consenting to B entering A’s home. It would be bad for A if B were permitted to enter A’s home whenever B wanted to. It would also bad for A if B were never permitted to enter A’s home, even when A would like B to enter. The normative power of consent enables A to determine when it is permissible for B to enter A’s home.^9^ It thereby makes it possible for A to ethically host a dinner party or a birthday party for friends. Put generally: we have a strong interest in being able to influence the normative status of others’ actions with regard to us. And given that this interest is not peculiar to some individuals, it suggests that all agents have this power of consent and they have it with respect to all other agents.

Still, there are limits on the normative power of consent. A classic example is slavery. A cannot consent to being enslaved by B in the sense that B’s enslaving A would be ethically permissible. A less classic example is Russian roulette. I cannot make it permissible for others to put a gun to my head, spin the barrel, and pull the trigger by consenting to being so treated. These limits are not explained by the fact that individuals can provide valid consent to slavery, but enslaving them is impermissible for other reasons—for example, because it has the potential to undermine public trust or order. Rather, the normative power of consent itself is limited: it cannot be exercised with respect to others enslaving us. Why, exactly, is the normative power of consent limited? Given that we have the power of consent, and given our strong interest in e.g. bodily autonomy, why can’t we make it permissible for others to enslave us, or expose us to the risks of Russian roulette?

Imagine a world in which we had the normative power to consent to anything and everything. In that world, we would be significantly worse off compared to the actual world. In other words, our interests are better promoted by living in a world in which our power of consent is limited. First, we are far from infallible reasoners. An unlimited power of consent would expose us to the possibility of making dramatic mistakes, to consenting to playing Russian roulette. Our fallibility suggests that we are better off with a power of consent that is limited, with one that cannot make it permissible for others to inflict the risks of Russian roulette on us.

Second, while the risks are high, the potential benefits of having an unrestricted power of consent are modest. It is only in the rarest cases, if any, that having the power to consent to being enslaved would promote our interests. Given the costliness of mistakes and the rarity of benefits, we are much better off in a world in which our power of consent is limited.

This line of reasoning suggests a justification which is internal to the nature of consent: an action cannot be made permissible by consent if we are better off with a normative power of consent that does *not* include the action in question (as opposed to having a normative power of consent that *does* give us this power). Call this the *Interest Principle*. What does it suggest with respect to research risks with consenting adults?

We noted earlier that minimal and a minor increase over minimal net risks are acceptable with participants who cannot consent. This raised the question of the extent to which competent adults can make greater risks acceptable by consenting to them. According to the Interest Principle, the answer depends on whether and to what extent we are better off having a power of consent that enables us to make greater risks permissible as opposed to a power of consent that does not include making greater risks permissible.

We noted earlier that a study which poses a 50% risk of death is unacceptable even with consenting adults. This judgment is vindicated by the Interest Principle: we are better off having a power of consent that does *not* allow us to make it permissible for others to impose a 50% risk of death on us for the benefit of others than in having a power of consent that allows us to do so. If we could make a 50% risk of death permissible, the harms that we would incur would outweigh the benefits. Put differently, the badness of a 50% chance of dying outweighs the benefit of having normative control over whether to incur a 50% chance of dying. Accordingly, the Interest Principle implies that a procedure with a 50% mortality rate poses too high a level of risk, even for consenting adults.

This conclusion depends on facts about the nature of our lives, decision-making capacities, and interactions with others. If we never made any mistakes—in the sense of reasoning poorly about what is good and bad for us, or what will or won’t lead to our getting what we want—it might be in our interests to have the broadest power of consent possible. However, we are not idealized reasoners, but finite and error-prone beings who make mistakes. We make mistakes with respect to evaluating our own interests, how often we can expect a certain degree of control to work to our benefit, and how often we can expect it not to.

This illustrates a point to which we will return: whether having the power to consent to an activity is in our interests depends critically on how broadly or narrowly we describe the activity in question. But first, it’s worth distinguishing the Interest Principle from a nearby but importantly different view, namely, from the view that we have the power to consent to all and only those activities that are in our (expected) interests. Put another way, if the point of having the normative power of consent is to promote our interests, why not think that our power of consent covers all and only those token activities which are ex ante in our interests?

In theory, the activities we could consent to range from maximally broad—any harm or interference—to maximally narrow—we can’t consent to others doing anything to us under any circumstances. Both the Interest Principle and this nearby competitor delimit the scope of the power of consent based on our interests: what we can consent to depends on what is in our interests. But they appeal to our interests in different ways. The Interest Principle holds that we can consent to a given activity just in case it is in our interests to have a power of consent that enables us to consent to activities with its net risks. The nearby competitor, on the other hand, holds that we can consent to a given activity just in case the activity is ex ante in our interests.

Which of these two views correctly describes the scope of the power of consent depends on which scope it is more in our interests to have. And the answer to that question is not determined by appeal to ideal and fully rational agents. Instead, it is determined by which approach better promotes the interests of actual human beings, taking into account our capacities as well as our fallibility.

The normative power of consent is valuable because it enables us to consent to a broad range of activities and, thereby, to choose how to live our lives. While a good deal more would need to be said to develop a full account, this description is sufficient to understand why the Interest Principle does not support permitting all and only the token consents which are in the interests of competent adults. First, this approach would imply implausibly that we cannot permissibly sacrifice our interests to any extent for the benefit of others. In the context of medical research, we would not have the power to consent to a clinical trial that poses minimal risks on us and offers significant potential benefit to others.^10^

Second, this view would entail that we have no freedom to make bad choices to any extent. Sometimes, we consent to others treating us in ways that are ultimately bad for us: we invite someone to a party when they foreseeably end up ruining the mood, or we buy a product at a price that is a bad deal. Human beings are not endowed with the capacity to accurately assess the precise consequences of every decision prior to making them, and we don’t always know whether consenting to something will ultimately work to our benefit. The fact that the risks outweigh the benefits in some cases does not invalidate our consent to them. If it did, it would be our interests, rather than ourselves, that are ultimately in control of our lives. In addition to its facial implausibility, this view ignores the fact that one of our strongest interests is our interest in determining the course of our lives for ourselves.

Third, given the variability in individuals’ ability to recognize what is in their interests, and anticipate the consequences of different courses of action, this view implies that the scope of the power of consent would vary widely across individuals. Some adults would have the power to consent to haircuts, others wouldn’t. Some would be able to choose where to go on vacation, but not which career to pursue or whom to date. Others which career to pursue, but not where to go on vacation.^11^

These concerns reveal that the interests-based approach does not support characterizing the scope of activities that competent adults can consent to in terms of all and only the token activities that are ex ante in our interests. But this still leaves open the question of exactly which activities *are* included within the scope of the power of consent that is most in our interests to have. To pursue this question, consider again the fact that we cannot make it permissible for others to expose us to the risks of Russian Roulette.

One might try to explain this limitation on the scope of consent by arguing that it would be contrary to our interests to be able to consent to others killing us. Historically, that was perhaps the dominant explanation, and some still endorse it. But advocates of euthanasia argue that it is in our interests to be able to consent to others killing us in certain circumstances. Whatever one thinks of this view, a general account of the normative power of consent should not preemptively reject it. To determine, then, what scope of power of consent is most in our interests, we need to specify the type of action in question. In the present case, we need to distinguish between consenting to others killing us when, say, we have a terminal illness and are suffering versus consenting to others killing us when we are happy and healthy. The fact that advocates of euthanasia endorse the former does not commit them to endorsing the latter.

A complete account of the interests-based approach would need to specify the level of generality at which our powers of consent are defined. While we don’t need to pursue that challenge in depth here, one point is critical for present purposes. In terms of the Interests Principle, there is no reason to think that the level of generality at which the permitted activities should be described will be constant across different types of activities. It might be that the interests of competent adults are best promoted by prohibiting us from consenting to all the subtypes of one activity (e.g. slavery), but prohibiting us from consenting to only some of the subtypes of another activity (e.g. others killing us). This reveals that we cannot address the present challenge of determining the limits on net research risks by appeal to some fixed level of generality. Instead, we will need to consider medical research specifically. However, as noted above, not everyone agrees that our possession of normative powers is explained in terms of our interests. Some hold instead that our possession of normative powers is explained by the ways in which they are bound up with our autonomy. Hence, before looking at the relation between the power of consent and limits on risks, we will outline the autonomy-based explanation.

Section 3: How Consent Justifies Risks: Autonomy and Respect

Autonomy-based accounts claim that we have certain normative powers because having this type of control over what happens to us is part of what makes us autonomous agents in the first place. This explanation starts from the claim that we are autonomous in some relatively robust sense, and then argues that (1) we could not or would not be autonomous in this sense if we did not have the normative power in question, or (2) our autonomy would lack its characteristic value or significance if we did not have the normative power in question.

One version of this view maintains that the value of autonomy lies in our having the broadest control over our lives possible. On this version, the value of autonomy implies that we have the power to consent to anything and everything. However, the fact that we do not have the normative power to consent to slavery or murder reveals that this view is mistaken. An alternative version of this approach maintains that the value of autonomy lies not in our having the broadest control over our lives possible, but in our having a sufficient level of control. Others appeal to more nuanced conceptions of autonomy, such as the “capacity for self-legislation.” (Hurd 1996, 124) While these versions seem more plausible, they are too vague to determine whether a given level of risk is too high to allow in clinical trials.

One way to try to make this approach more precise is to appeal to the broader concept of respect. Autonomy and respect share an intimate connection: we have a moral obligation to treat others with respect. And respect for other people requires respecting their autonomy. In other words, treating autonomous individuals as though they lack the power of consent would violate our duties of respect to them. In the words of the Belmont Report: “The principle of respect for persons thus divides into two separate moral requirements: the requirement to acknowledge autonomy and the requirement to protect those with diminished autonomy.” This approach suggests that the way to determine what the normative power of consent encompasses is to consider what respect requires of us when it comes to the autonomy of others.

Consider A consenting to B entering their home, once again. A has the normative power to make it permissible for B to enter A’s home, without which it is impermissible for B to enter A’s home. But rather than explaining this normative power in terms of its promoting people’s interests to have control over who is allowed to enter their homes, an autonomy-based explanation holds that A has this normative power because denying A this power—that is, treating A as though they lacked this power—would disrespect A, or violate our duty of respect toward A. Part of what is involved in treating others with respect—or treating them as autonomous agents—is granting them authority over their lives, including whether and how others use their property. And having the normative power to permit others to enter one’s home is essential to having this kind of authority.

One might think that it is always more respectful to accord others greater control over their lives. If that is right, individuals would have the normative power to consent to being enslaved. However, part of why it is wrong to enslave (murder, torture) others, even when they (attempt to) consent to it, is that the act of doing so would itself be disrespectful to them. In other words, giving others control over their lives is only part of what is involved in treating them with respect. Torturing another person, for instance, fails to respect them even when they agree to being so treated. Treating others with respect is in part a matter of taking their morally significant interests seriously, whether the interest in question is an interest in controlling the course of their lives or something else, such as bodily integrity. Accordingly, when someone expresses a desire to incur significant risks or harms, acceding to their wishes would often disrespect them by failing to treat their basic interests as worthy of protection.

Given that duties of respect require us to treat one another as independent agents with the authority to determine what happens to them, it may seem that to treat someone with respect involves treating them as having a maximally wide scope of decisional authority. And accordingly, it may seem that to be treated as lacking the option of consenting to being e.g. enslaved is to be treated with disrespect, and so a respect-based approach to consent cannot justify restricting the power of consent in the case of slavery. But this appearance is misleading. Duties of respect require us to treat one another as independent agents because they require us to weigh one another’s morally significant interests with proper regard, and we have a morally significant interest in being able to live our lives as independent agents. But while this interest is weighty, it is also just one interest among many, and does not automatically trump others.^12^ Put differently, while being an autonomous agent is an important part of who we are, it is not everything. Hence, respect for us goes beyond respect for our autonomy. Part of respecting someone is weighing their interests with proper regard, and this may be to treat them as unable to consent to harmful or risky activities, rather than as having a maximally broad-scope power of consent. So like the interests-based approach, the respect-based approach supports internal limits on the power of consent.

According to the respect-based approach, then, the power of consent has limits because treating someone as though they were able to consent to severe risks would fail to treat them with respect. Importantly, this claim has a *ceteris paribus* or “all else equal” character. The question of whether some form of treatment would be respectful or disrespectful is sensitive to a range of considerations, including the context in which the action occurs, the personal values at stake, and the relevant alternatives. Some individuals—for instance, skydivers and rock climbers—derive great meaning and personal value from high-risk activities. For many, there are no less-risky alternatives that could provide the same personal value, and so it may be disrespectful to deny them the opportunity to incur these risks. But the situation is typically different in medical research. Participants do not enroll in research in search of a thrill, and there are many opportunities for low- and lower-risk altruistic activities besides high-risk medical research. Further, even if there are a small number of participants with respect to whom imposing severe research risks would not be disrespectful because of their personal values and alternatives, this provides little reason to abandon risk limits in research regulations, since doing so would involve treating many other potential participants with disrespect.

By using this reasoning about the internal limits on the power of consent according to the respect-based view, then, we can formulate a principle concerning limits on the harms or interferences that can be made permissible by consent, analogous to the Interest Principle. Namely, some harm or interference cannot be made permissible by consent if treating someone as though they had the power to consent to it would be to treat them disrespectfully, or to violate a duty of respect to them. We will call this the *Respect Principle*. Like the Interest Principle, the Respect Principle delimits the types of harms or interferences that we can make permissible through the power of consent. And like the Interest Principle, the Respect Principle says that we have a substantial and strong power of consent, but one that does not extend to being treated in significantly harmful or risky ways with no potential for compensating benefit. The difference between the Interest Principle and the Respect Principle lies not in the harms or interferences that they allow and rule out, but rather in their explanation for why these harms or interferences are allowed or ruled out.

Regardless of whether we adopt an interests-based or respect-based explanation of the normative power of consent, then, we can evaluate the limits on how much risk can be made permissible by consent by appeal to the nature of consent as a normative power. Accordingly, to determine the limits on acceptable risks for consenting adults, we must look at what levels of risk could be made permissible by either (1) the power of consent that we have the strongest interest in possessing, or (2) the power of consent that would best accord with our duties of respect to one another.

The preceding considerations suggest that the power of consent allows a similar level of risk on either explanation. But it is difficult to say with any precision what level this would be in the abstract—especially when aggregating risks of harms at various levels of severity. Determining where the limit lies involves balancing our interest in controlling how others treat us with our interest in not being significantly harmed or interfered with. And further, in order to make this determination with any significant degree of specificity, we would have to know all the contexts in which it would be in our interests to have this power, the benefits we would get from each of them, and even the probabilities of finding ourselves in each of these contexts. This determination cannot be made in the abstract. Instead, we will show how these abstract considerations about the nature of consent can guide decision-making in particular circumstances. To this end, we turn to a particular question of clinical importance, and look at what form of normative power of consent would be in our interests in this case: namely, the question of the permissibility of performing research kidney biopsies on consenting adults. By looking at how consent works in this case, we will illustrate how either version of the consent-based approach to risk limits can be applied to evaluate specific risks in real-world contexts.

Section 4: The Implications for Kidney Biopsy Research

Kidney disease is very common. Acute kidney injury, for instance, affects over 13 million people worldwide every year (Lamiere et al. 2013), and roughly 37 million Americans have chronic kidney disease (CDC 2022). Researchers looking to develop new medicines and treatments rely on kidney biopsies from those with kidney disease as well as healthy individuals. The risk of death from kidney biopsy is approximately 1–3 in 1000 procedures to 1 in 7000 or 8000 procedures (Halimi 2020, Koirala 2020, Poggio 2020).

On the Interest Principle, whether it is permissible to impose this risk on consenting adults depends on whether is it in our interests to have a normative power of consent that includes these risks. On the Respect Principle, it depends on whether according others the power to consent to these risks involves treating them with respect or not. To make this determination, we need to identify the *consent-relevant description* of the risks associated with kidney biopsies. Is the consent-relevant description: the medical procedure along with its associated risks—having a kidney biopsy along with the risk of death? Or is it: the net level of risk, plus the fact that the harm or interference would occur in the context of medical research? Or simply: the net level of risk, regardless of the exact harms and frequencies that constitute it?

The strength of our interest in not being subjected to the risk of a particular harm (in this case, significant bleeding and death) depends on the magnitude of the net risk, not on the specific type of harm in question. Moreover, we sometimes have a stronger interest in not being subjected to risks in one domain than in others. For instance, our interest in our right to privacy not being violated is stronger when dealing with representatives of the state than when interacting with friends. However, this is because the net risks involved in some domains are higher than those in others.^13^ This suggests that what matters to whether we can consent to some harm or interference is the net risks associated with it.^14^ It follows that the consent-relevant description of the harms is that of the net risks.

We are now in a position to assess whether the risks involved in kidney biopsies exceed the ethical limits on risk for consenting adults. The question is whether we have a stronger interest in, or our duties of respect better accord with, having (1) a normative power of consent that gives us the power to make it permissible for others to impose the net risks involved in excessive bleeding and death associated with kidney biopsies on us; or (2) a normative power of consent that prevents us from making it permissible for others to impose these risks on us. And here the answer is, we think, relatively clear. Although it is by default impermissible for others to impose these risks on us, (1) we have a strong interest in being able to make it permissible for others to do so, and (2) it would be disrespectful to treat someone as though they lacked the authority to consent to these risks.

We have a strong interest in being able to make sacrifices to help those we love, such as when we donate organs. We have a strong interest in being able to dedicate our lives to helping others in dangerous situations, such as when we become firefighters. We even have a strong interest in being able to take significant risks in the hope of personal gain, as when we allow someone to invest our money for us. Undertaking even significant risks in order to help others is, at least for many people, tremendously important.^15^ Many research participants feel their lives would be worse if they could not engage in such pursuits, and being prevented from doing so would be significantly disrespectful, even an affront to their authority over their lives. None of these activities are mandatory, but they all may involve others imposing significant risks on us. Insofar as we think that individuals have a sufficiently strong interest in being able to pursue these sorts of goals, we think that we have a stronger interest in having, and can only be treated with respect when treated as having, the first type of normative power of consent: a normative power of consent that enables us to make it permissible for others to impose the net risks associated with kidney biopsies on us.^16^

In particular, the risks of excessive bleeding and death associated with kidney biopsies can be directly compared with the risks associated with kidney donation. The risk of death associated with kidney donation is roughly 0.03-0.06%, while kidney biopsy is estimated to have a 0.0125-0.3% risk of death (and, notably, this estimate for the risk of death associated with kidney biopsy is based on individuals with kidney disease rather than healthy individuals, for whom the risk of death could conceivably be lower). As we noted above, it neither is nor should be the job of moral theory to determine the precise levels of risk that are acceptable in these contexts, since making a complete determination requires the judgments of reasonable and informed individuals. What moral theory can do is to provide a framework that explains why there are such limits, and what considerations go into concrete determinations of the acceptability of risks. And both of the consent-based approaches to risk limits that we have outlined can do exactly that. Insofar as we regard the normative power of consent as allowing individuals to make it permissible for others to impose the risks associated with kidney donation on them, we should likewise regard the normative power of consent as allowing individuals to make it permissible for others to impose the risks associated with kidney biopsy on them.

A final point in favor of the consent-based approach to risk limits lies in its ability to explain the difference in ethically acceptable risk limits between consenting adults and those who lack the capacity to consent. Regulations for pediatric research, for instance, allow both minimal and a minor increase over minimal levels of risk.^17^ And while these regulations lack precise measures for what constitutes a minimal or minor increase over a minimal level of risk, a popular approach suggests that these levels should be understood in terms of the risks that children encounter in daily life, where these can include small but nonzero risks of death. The consent-based approach to risk limits is not only consistent with greater restrictions on the risks that can be imposed on children, but further, it provides an explanation for why they lack the capacity to consent to higher risks. In terms of the Interest Principle, children have a weaker interest in being able to make harms to them permissible through consent, and their interest in welfare is plausibly even more threatened by severe risks than consenting adults’ interest in welfare. And in terms of the Respect Principle, the duties of respect we owe children involve placing less weight on their interest in having the authority to determine what happens to them, and more weight on their interest in being protected from severe harms.

According to the consent-based approach to risk limits, then, the explanation for why risks in pediatric research should be significantly restricted (in order to protect those who cannot consent) goes hand in hand with the explanation for why consenting adults can be exposed to significantly higher levels of risk. Implicit in restrictions on risks like those in pediatric research regulations is the view that those who have the capacity to consent may accept significantly higher levels of risk—lest the pediatric regulations provide no significant protection.

Regardless of whether we use the Interest Principle or Respect Principle, the consent-based approach to risk limits differs substantially from utilitarian views that understand the acceptability of research risks in terms of simply weighing the potential harms to participants against the social benefits that research may produce. While understanding risk limits in terms of consent does grant that harms to participants and social benefits of research are relevant to the acceptability of research risks, an approach that simply weighs harms against benefits implies that there are no risks too large to impose on participants, so long as the benefits are large enough as well.

Our approach to risk limits starts by asking how much risk is acceptable without consent and then working forwards to find out how much risk consent can justify. It is best pursued on a case-by-case level, by looking at the particular risks associated with some procedure or intervention and asking about their relation to the normative power of consent itself. The question we must ask ourselves, in determining how much risk can be imposed on research participants, is what kind of normative power of consent best serves our interests and coheres with the duties of respect that we are owed.

## Footnotes

1 See CIOMS 2016, Guideline 16.

2 A notable exception, which largely proves the rule, is the Nuremberg Code, which states: “No experiment should be conducted where there is an a priori reason to believe that death or disabling injury will occur; except, perhaps, in those experiments where the experimental physicians also serve as subjects.”

3 Importantly, our view also suggests that a study with these risks would be unacceptable even as the benefits become arbitrarily large: even if such a study stood a 99% chance of saving the lives of billions of people, our view suggests that it would impose unacceptable levels of risk on its participants. While such cases may seem to pose a difficulty for our view, we note that our account is designed to make sense of risk limits in the conduct of actual medical research, and such hypothetical cases stand far removed from anything resembling the actual practice of medical research. What is ethically acceptable may change in dire emergencies, and in such cases, we may be required to act in ways that would be unacceptable in most real-world circumstances.

4 Eyal (2022) notes that appeals to public trust are ambiguous between an empirical reading, which is factually dubious, and a normative reading, which is question-begging against those who oppose the risk limit in question.

5 See US Code 45 CFR 46.404.

6 There is a literature in bioethics on the question of whether the determination of decision-making capacity should be sensitive to the risks involved in the procedure or study under consideration. (See, for instance, Buchanan and Brock 2019, Bromwich and Rid 2015, Kim and Berens 2023) While the views in this literature do not directly answer the question of limits on research risks, they may be taken to bear on how to account for risk limits. In particular, if the determination of decision-making capacity should be risk-sensitive, then there may be certain levels of risk concerning which no individuals should be deemed to have decision-making capacity. Such a view would bear some similarity to ours in virtue of focusing on the conditions of valid consent. However, by contrast, our argument below does not rely on any claims concerning competence or decision-making capacity in particular.

7 US regulations for pediatric research also contain a category, 45 CFR 46.407, which does not have an explicit risk limit, but is rarely used and requires approval from the Secretary of the U.S. Department of Health & Human Services.

8 There is debate among moral philosophers over precisely which conditions must be satisfied in order to exercise a normative power more generally. For present purposes, we will focus on two conditions that are endorsed on most accounts: (1) intending to change the normative status of an action; and (2) communicating that intention, typically to the person whose action is at issue.

9 Of course, A doesn’t necessarily have the same degree of interest in making it permissible for every individual to enter their home: they presumably have a much stronger interest in allowing a friend than a serial killer to enter. This raises the question of the level of specificity with which we should describe the actions being consented to, and we return to this question below. But suffice it to say for now that whether or not we have the power to consent to some action or interference is universal across agents in the sense that if one agent has this power then *every* agent has it, and if an agent has the power to make it permissible for someone to impose some harm or interference on them then they have the power to make it permissible for *anyone* to impose that harm or interference on them.

10 Indeed, one might worry that the Interest Principle would rule out cases of noble self-sacrifice, such as when one person jumps onto a grenade to save two of their comrades in arms. Doing so strikes us as not only permissible but laudable. But the Interest Principle does not rule out self-sacrifice; it rules out one person’s ability to make it permissible for another person to shove them onto a grenade. Not everything that we may permissibly do to ourselves is something that we can make it permissible for others to do to us. Note also the point in fn. 3: we are aiming to account for risk limits in research regulations for actual medical practice, and what is ethically acceptable in dire emergencies may be different. It is one thing to attempt to enlist another person’s help in sacrificing oneself, and another for research regulations to permit participants to incur levels of risk in standard practice that amount to self-sacrifice.

11 It’s worth noting that precisely how we apply the Interest Principle to different cases will also depend on how we assess what is in our interests, and so how to assess wellbeing. In particular, some views of wellbeing may hold that the feeling of constraint that may occur when we lack the ability to consent to some activity is a detriment to one’s wellbeing, while other views may restrict the scope of wellbeing to the outcomes of our decisions. But the differences between such views of wellbeing do not make a difference to the application of the Interest Principle to the central cases we discuss, and so we refrain from committing to any particular view of interests or wellbeing.

12 Note also that in the case of enslavement, the very interest in independence that normally makes it disrespectful to disregard someone’s wishes may point precisely in the other direction.

13 Of course, participants may have a stronger reason to want to incur some level of risk in a biomedical context than in others—for instance, if they have a personal connection to some disease and want to help current and future individuals who it affects. But the strength of individuals’ reasons to want to incur some level of risk speaks to the rationality of exercising their power of consent, if they in fact have the ability to consent to risks of the relevant strength, not to whether they have the power of consent in the first place. The Interest Principle and Respect Principle consider the strength of our reasons in favor of having a power of consent that makes us able to consent to given levels of risk, not the strength of our reasons in favor of exercising such a power.

14 One might have the following objection at this point: isn’t the fact that the participants in a clinical trial stand to create significant benefits to others relevant to the risks they may incur? If we simply adopt a net risk approach, then the limits on the risks an individual can consent to remain the same regardless of whether they would be generating any benefits to others by incurring the risks. And this would seem to stand at odds with the way in which the social benefits of clinical research are relevant to the permissible risks to participants. But this objection fails to distinguish between two related but distinct criteria for ethical research: first, the risks must be justified by benefits to participants or others, and second, the risks must not be too severe in themselves. We agree with this objection that the fact that research creates social benefits is relevant to the risks that participants may incur. But its relevance is captured by the first criterion, and our focus in this paper is the second. Social benefits are relevant to permissible risks because any risks to participants must be justified by benefits—not because social benefits help to determine the maximum limits on risks to participants themselves.

15 On altruistic participation in clinical research, see (Jansen 2009), (Truong 2011), (Olsen 2020).

16 Note again that in asking whether some clinical trial exceeds the ethical limits on risks, we do not compare the personal benefits of engaging in the altruistic activity of participating with the burdens associated with the risks of the trial. Instead, we compare the personal benefits (or the cohesion with our duties of respect) of having a power of consent that allows individuals to consent to the net risks of the trial *irrespective of context* (i.e. not just the benefits that accrue in the context of the trial) with the burdens associated with having such a power of consent, in light of our liability to reason poorly, plurality of personal values, and variety of contexts and alternatives. In making this comparison, we do not rely on a mechanical decision procedure, but instead look to our practices of allowing and forbidding similarly risky activities for guidance regarding what levels of risk are permitted by the lights of the Interest Principle and Respect Principle.

17 For instance, we may estimate the risk of death consistent with a minimal risk standard at around 1 in 300,000. If so, then a risk of death of 1 in 100,000-150,000 would represent a minor increase over minimal risk, while a risk increase of an order of magnitude or larger, such as 1 in 30,000, would be greater than a minor increase over minimal risk. See (Rid et al., 2010).
